# Protective effects of inulin against lipopolysaccharide-induced oxidative stress in broiler chickens

**DOI:** 10.3389/fvets.2025.1660935

**Published:** 2025-10-28

**Authors:** Qinghui Shang, Hao Li, Yan Han

**Affiliations:** Shandong Provincial Key Laboratory of Livestock and Poultry Breeding, Poultry Institute, Shandong Academy of Agricultural Sciences, Jinan, China

**Keywords:** inulin, broiler, lipopolysaccharide, oxidative stress, antioxidant capacity

## Abstract

**Introduction:**

This study was conducted to evaluate the protective effects of inulin against lipopolysaccharide (LPS)-induced oxidative stress in broilers.

**Methods:**

A total of 108 1-day-old male Arbor Acres broilers were randomly allocated to 3 treatment groups with 6 replicates per group and 6 birds per replicate. The 3 groups were: (1) non-challenged broilers fed a corn-soybean meal basal diet (control group, CON); (2) LPS-challenged broilers fed the basal diet (LPS); (3) LPS-challenged broilers fed the basal diet supplemented with 15 g/kg inulin (LPS + Inulin).

**Results:**

The results showed that LPS administration inhibited activities of antioxidant enzymes and stimulated production of lipid peroxidation in multiple tissues including serum, liver, intestine, and muscle. Dietary inulin supplementation partially alleviated the negative effects of LPS on antioxidant capacity of these tissues, with the underlying mechanism involving the regulation of the Nrf2 signaling pathway.

**Discussion:**

Collectively, these findings indicate that inulin supplementation effectively alleviates LPS-induced oxidative stress, highlighting its potential as a feed additive in broiler diets to combat oxidative stress.

## 1 Introduction

Over the past decades, intensive genetic selection for fast-growing strains has remarkably optimized the productivity of commercial broiler chickens (1). Meanwhile, however, unbalanced genetic selection for production traits over immune traits has increased susceptibility to stressors in broiler chickens, thereby elevating their risk of diseases (2). Unfortunately, broiler chickens are subjected to a diverse range of stressors in modern intensive production, including bacterial infection, mycotoxins, heat stress, high stocking density, etc. (3). These stressors can trigger oxidative and immunological stress, thereby negatively impacting overall health and growth in broilers (4). Lipopolysaccharide (LPS), also known as endotoxin, is a central component of the cell wall of Gram-negative bacteria and is widely recognized as one of the most prevalent stressors in production (5). It has been demonstrated that LPS exposure can induce systemic inflammation, disrupt intestinal function, compromise growth performance or even lead to death, thereby causing considerable economic losses (6, 7). In addition, the move away from in-feed antibiotic growth promotors has rendered it more difficult to help broilers cope with LPS challenge. Therefore, it is of paramount importance to develop safe and efficient strategies to mitigate the adverse effects of LPS on broiler chickens.

Nutritional manipulation has shown to be effective in addressing LPS challenge and maintaining the health and performance in broilers (8, 9). Inulin is a natural polydisperse polysaccharide belonging to the fructan family, composed of linear chains of fructose molecules with a terminal glucose (10). It has shown to provide numerous health benefits, including modulating the immune system, inhibiting inflammation, enhancing antioxidant capacity, regulating gut microbiota, promoting mineral absorption, and improving lipid and sugar metabolism (11, 12). Previous research indicated that dietary supplementation of inulin could improve the gut microbiota, enhance immune function, and promote growth performance in broilers under normal conditions (13). Furthermore, our recent study suggested that inulin alleviated LPS-induced inflammation in broilers, exhibiting a great potential to deal with LPS challenge (14). However, oxidative stress has been recently identified as a pivotal factor in the initiation and progression of LPS-induced challenges in broiler chickens (15). Growing evidence suggests that mitigating oxidative stress seems to be a key mechanism through which many nutritional strategies alleviate LPS-induced afflictions in broiler chickens (16). Although the effects of inulin on LPS-induced inflammation have been investigated, its role in LPS-induced oxidative stress still remains unclear. Considering the antioxidant properties of inulin, it is hypothesized that inulin could alleviate LPS-induced oxidative stress. Therefore, the aim of this study was to explore the effects of dietary inulin supplementation on antioxidant capacity in multiple tissues of LPS-challenged broiler chickens. The findings will contribute to a more comprehensive understanding of the mechanism through which inulin mitigates LPS-induced negative effects.

## 2 Materials and methods

### 2.1 Ethical approval

All experimental protocols were approved by the Institutional Animal Care and Use Committee of Shandong Academy of Agricultural Sciences (Jinan, China; SAAS-2024-G24).

### 2.2 Birds, house and management

A total of 108 1-day-old male Arbor Acres broiler chickens were procured from a local commercial hatchery and reared in the poultry house of Poultry Institute, Shandong Academy of Agricultural Sciences (Jinan, China). Birds were raised in three-tiered cages in an environmentally controlled house. Each cage (90 × 60 × 40 cm) was equipped with a feeder and a nipple drinker to provide free access to feed and water. The temperature was maintained at 33 °C during the first week, followed by a gradual reduction of 3 °C per week until reaching 24 °C. Relative humidity was maintained at 60 to 70% for the first 3 days and thereafter maintained at 50 to 60%. The light regimen was 23 h of light and 1 h of dark throughout the experimental period.

### 2.3 Experimental design

Upon arrival, chicks were weighed and randomly allocated to 3 treatment groups with 6 replicates per treatment and 6 birds per replicate. The 3 groups were: (1) non-challenged broilers fed a corn-soybean meal basal diet (control group, CON); (2) LPS-challenged broilers fed the basal diet (LPS); (3) LPS-challenged broilers fed the basal diet supplemented with 15 g/kg inulin (LPS + Inulin). The LPS from *E. coli* serotype O55: B55 was purchased from Sigma-Aldrich (L-2880; St. Louis, MO, USA). Inulin (90% purity) with an average polymerization degree ≥ 20 was obtained from Beneo Orafti (Tienen, Belgium). The basal diet was formulated to meet or exceed the nutrient requirements recommended by both the NRC (1994) and Arbor Acres, and was fed in mash form (Table 1). At 21 d of age, LPS-challenged broilers were administered an intraperitoneal injection of LPS solution at a dose of 5 mg/kg BW according to the previous study (13). Meanwhile, broilers in the CON group were injected with an equal volume of 0.9% sterile saline.

**Table 1:** Ingredients and nutrient levels of the basal diet (as fed basis, %).

Item	Content
Ingredients	
Corn	58.65
Soybean meal	30.39
Corn gluten meal	2.00
Fish meal	2.00
Soybean oil	3.20
Dicalcium phosphate	1.50
Limestone	1.30
Salt	0.30
L-Lysine HCl	0.01
DL-Methionine	0.14
L-Threonine	0.01
Premix^1^	0.50
Nutrient levels^2^	
Metabolizable energy, MJ/kg	12.77
Crude protein	21.01
Calcium	1.00
Total Phosphorus	0.70
Available Phosphorus	0.45
Lysine	1.10
Methionine	0.50
Threonine	0.80
Tryptophan	0.28

^1^Premix supplied per kilogram of diet: vitamin A, 11000 IU; vitamin D, 3025 IU; vitamin E, 22 mg; vitamin K_3_, 2.2 mg; thiamine, 1.65 mg; riboflavin, 6.6 mg; pyridoxine, 3.3 mg; cobalamin, 17.6 μg; nicotinic acid, 22 mg; pantothenic acid, 13.2 mg; folic acid, 0.33 mg; biotin, 88 μg; choline chloride, 500 mg; iron, 48 mg; zinc, 96.6 mg; manganese, 101.76 mg; copper, 10 mg; selenium, 0.05 mg; iodine, 0.96 mg; cobalt, 0.3 mg.

^2^Nutrient levels are calculated values.

### 2.4 Sample collection

After 4 h of LPS exposure, 1 bird per replicate cage (6 birds/group) was selected. Blood samples were collected from wing vein and coagulated for 30 min at room temperature. Then serum was obtained by centrifugation at 3000 × g for 15 min at 4 °C and stored at −80 °C until analysis. After then, birds were euthanized humanely. Jejunal section was flushed with 0.9% ice-cold saline, then the mucosa was scraped with a sterile glass slide, snap frozen in liquid nitrogen and stored at −80 °C. Liver, breast and thigh meat samples at the same position were also collected, snap frozen in liquid nitrogen and stored at −80 °C until further analysis.

### 2.5 Antioxidant capacity analysis

Antioxidant capacity in the serum, jejunum, meat and liver including superoxide dismutase (SOD, No. A001-1), glutathione peroxidase (GSH-Px, No. A005-1), catalase (CAT, No. A007-1), malondialdehyde (MDA, No. A003-1) and total antioxidant capacity (T-AOC, No. A015-3) was measured by commercial kits (Nanjing Jiancheng Biotechnology Institute, China) following the manufacturer’s instructions. Total protein concentration in the jejunal mucosa, meat and liver was measured using a bicinchoninic acid protein assay kit (Beyotime, Shanghai, China). Values were expressed as units/g protein.

### 2.6 Real-time qPCR analysis

Hepatic mRNA expression of nuclear factor erythroid-derived 2-related factor 2 (Nrf2) and Kelch-like ECH-associated protein 1 (Keap1) were determined by using real-time quantitative PCR. Total RNA was extracted from the liver samples using TRIZOL reagent (Invitrogen, Carlsbad, CA) according to the manufacturer’s instructions. The quality and quantity of RNA was determined using a NanoDrop 2000 spectrophotometer (Thermo Scientific, Wilmington, USA). The integrity of RNA was determined using agarose gel electrophoresis. Then the RNA was reverse transcribed into cDNA using a PrimeScript® RT reagent kit with gDNA Eraser (TaKaRa, Dalian, China).

Real-time qPCR was performed using LightCycler® 480 II Real-time PCR Instrument (Roche, Swiss) with 10 μL PCR reaction mixture containing 1 μL of cDNA, 5 μL of 2 × PerfectStartTM Green qPCR SuperMix, 0.2 μL each of forward and reverse primers and 3.6 μL of nuclease-free water. The thermal cycling conditions were as follows: pre-denaturation (30 s at 94 °C); 45 cycles of amplification (5 s at 94 °C and 30 s at 60 °C). Each sample was run in triplicate for analysis. At the end of the PCR cycles, melting curve analysis was performed to validate the specific generation of the expected PCR product. The primer sequences used in this study were referenced from previous literature (17) and synthesized by TsingKe Biotech (Beijing, China). Relative mRNA expression was normalized to GAPDH and was calculated using the 2^–ΔΔCt^ method.

### 2.7 Statistical analysis

All analyses were conducted using SAS 9.2 software (SAS Inst., Inc., Cary, NC). Data normality was evaluated by the Shapiro–Wilk test. For the normally distributed data, one-way analysis of variance (ANOVA) followed by Tukey’s test was employed. For the non-normally distributed data, the Kruskal–Wallis test was used. *p* < 0.05 was considered statistically significant.

## 3 Results

### 3.1 Serum antioxidant capacity

The effects of inulin on serum antioxidant capacity of LPS-challenged broilers are shown in Table 2. The LPS injection significantly decreased (*p* < 0.05) serum SOD, GSH-Px and CAT activities, and increased (*p* < 0.05) serum MDA production when compared to the CON group. Supplementation of inulin markedly prevented (*p* < 0.05) the LPS-induced decrease in serum SOD activity as well as the increase in serum MDA level, with both parameters nearly reverting to baseline levels observed in the CON group. However, no significant differences in serum GSH-Px and CAT activities were found neither between the LPS and LPS + Inulin groups nor between the CON and LPS + Inulin groups. With respect to serum T-AOC activity, all three groups differed significantly (*p* < 0.01) from each other. The highest value was found in the CON group, while the lowest value was recorded in the LPS group, with the intermediate value being observed in the LPS + Inulin group.

**Table 2:** Effects of inulin on serum antioxidant capacity of LPS-challenged broilers.

Items	CON	LPS	LPS + Inulin	SEM	*p*-value
SOD, U/mL	60.64^a^	51.06^b^	56.11^a^	1.33	<0.01
GSH-Px, μmol/L	17.45^a^	13.69^b^	14.43^ab^	0.97	0.03
CAT, U/mL	8.53^a^	6.85^b^	7.03^ab^	0.41	0.02
MDA, nmol/mL	1.18^b^	1.40^a^	1.24^b^	0.04	0.01
T-AOC, U/mL	16.47^a^	10.20^c^	13.44^b^	0.55	<0.01

CON, non-challenged broilers fed the basal diet; LPS, lipopolysaccharide-challenged broilers fed the basal diet; LPS + Inulin, lipopolysaccharide-challenged broilers fed the basal diet supplemented with 15 g/kg Inulin; SOD, superoxide dismutase; GSH-Px, glutathione peroxidase; CAT, catalase; MDA, malondialdehyde; T-AOC, total antioxidant capacity; ^a-c^Means within the same row with different superscripts are significantly different (*p* < 0.05).

### 3.2 Jejunal antioxidant capacity

The effects of inulin on jejunal antioxidant capacity of LPS-challenged broilers are presented in Table 3. In comparison to the CON group, jejunal activities of SOD, GSH-Px, CAT and T-AOC were significantly reduced (*p* < 0.05) while jejunal MDA level was significantly increased (*p* < 0.05) after LPS administration. Non-significant improvements were detected in jejunal activities of SOD, GSH-Px and CAT by inulin supplementation when compared to the LPS group. However, jejunal MDA level decreased significantly (*p* < 0.05) in response to inulin supplementation compared to the LPS group, but was still higher (*p* < 0.05) than that in the CON group. Dietary inulin supplementation effectively inhibited (*p* < 0.05) the LPS-induced decline in jejunal T-AOC activity, leading to values comparable to the CON group.

**Table 3:** Effects of inulin on jejunal antioxidant capacity of LPS- challenged broilers.

Items	CON	LPS	LPS + Inulin	SEM	*p*-value
SOD, U/mg	50.03^a^	41.95^b^	44.65^ab^	1.58	0.01
GSH-Px, μmol/g	15.99^a^	12.94^b^	14.53^ab^	0.77	0.04
CAT, U/mg	26.35^a^	21.91^b^	24.25^ab^	0.99	0.02
MDA, nmol/mg	1.16^c^	1.42^a^	1.27^b^	0.02	<0.01
T-AOC, U/mg	14.73^a^	11.04^b^	13.22^a^	0.50	0.01

CON, non-challenged broilers fed the basal diet; LPS, lipopolysaccharide-challenged broilers fed the basal diet; LPS + Inulin, lipopolysaccharide-challenged broilers fed the basal diet supplemented with 15 g/kg Inulin; SOD, superoxide dismutase; GSH-Px, glutathione peroxidase; CAT, catalase; MDA, malondialdehyde; T-AOC, total antioxidant capacity; ^a, b^Means within the same row with different superscripts are significantly different (*p* < 0.05).

### 3.3 Meat antioxidant capacity

The effects of inulin on meat antioxidant capacity of LPS-challenged broilers are given in Table 4. Comparing the breast meat results obtained in the LPS group with those in the CON group, the activities of SOD and T-AOC were significantly reduced (*p* < 0.05). Supplementation of inulin non-significantly enhanced the activities of SOD and T-AOC, with values being similar to the CON group. No significant differences were observed in the activities of GSH-Px and CAT, as well as the level of MDA among the three experimental groups. For thigh meat, the LPS administration significantly decreased (*p* < 0.05) SOD activity, and elevated (*p* < 0.05) MDA level when compared to the CON group. Dietary inulin supplementation non-significantly promoted SOD activity, and significantly lowered (*p* < 0.05) MDA production, restoring both to the levels of the CON group. However, the activities of GSH-Px, CAT and T-AOC showed no significant differences among the three groups.

**Table 4:** Effects of inulin on meat antioxidant capacity of LPS-challenged broilers.

Items	CON	LPS	LPS + Inulin	SEM	*p*-value
Breast meat					
SOD, U/mg	47.01^a^	41.23^b^	45.87^ab^	1.55	0.04
GSH-Px, μmol/g	18.57	17.02	17.85	0.52	0.14
CAT, U/mg	26.79	23.74	24.58	1.42	0.32
MDA, nmol/mg	1.22	1.43	1.31	0.06	0.11
T-AOC, U/mg	12.45^a^	9.12^b^	10.06^ab^	0.87	0.04
Thigh meat					
SOD, U/mg	46.82^a^	41.85^b^	45.32^ab^	1.20	0.03
GSH-Px, μmol/g	19.68	17.34	18.13	0.77	0.12
CAT, U/mg	28.33	25.20	26.24	1.50	0.35
MDA, nmol/mg	1.19^b^	1.51^a^	1.26^b^	0.06	0.01
T-AOC, U/mg	11.91	9.39	10.33	0.97	0.21

CON, non-challenged broilers fed the basal diet; LPS, lipopolysaccharide-challenged broilers fed the basal diet; LPS + Inulin, lipopolysaccharide-challenged broilers fed the basal diet supplemented with 15 g/kg Inulin; SOD, superoxide dismutase; GSH-Px, glutathione peroxidase; CAT, catalase; MDA, malondialdehyde; T-AOC, total antioxidant capacity; ^a, b^Means within the same row with different superscripts are significantly different (*p* < 0.05).

### 3.4 Hepatic antioxidant capacity

The effects of inulin on hepatic antioxidant capacity of LPS-challenged broilers are listed in Table 5. The LPS challenge significantly decreased (*p* < 0.05) the activities of SOD, CAT and T-AOC, and markedly stimulated (*p* < 0.05) MDA formation in the liver compared to the CON group. Inulin significantly mitigated (*p* < 0.05) LPS-induced reduction in hepatic SOD activity, restoring it to the baseline level observed in the CON group. Although hepatic T-AOC activity was significantly enhanced (*p* < 0.05) in the LPS + Inulin group compared to the LPS group, it remained significantly lower (*p* < 0.05) than that in the CON group. Supplementation of inulin slightly mitigated LPS-induced decrease in CAT activity and increase in MDA level, but the differences were not significant. The GSH-Px activity did not differ among the three groups.

**Table 5:** Effects of inulin on hepatic antioxidant capacity of LPS-challenged broilers.

Items	CON	LPS	LPS + Inulin	SEM	*p*-value
SOD, U/mg	49.83^a^	43.11^b^	48.64^a^	1.38	0.01
GSH-Px, μmol/g	18.07	16.11	16.89	0.67	0.15
CAT, U/mg	38.63^a^	34.72^b^	36.60^ab^	0.98	0.04
MDA, nmol/mg	1.48^b^	1.83^a^	1.60^ab^	0.08	0.04
T-AOC, U/mg	11.56^a^	8.18^c^	9.86^b^	0.32	<0.01

CON, non-challenged broilers fed the basal diet; LPS, lipopolysaccharide-challenged broilers fed the basal diet; LPS + Inulin, lipopolysaccharide-challenged broilers fed the basal diet supplemented with 15 g/kg Inulin; SOD, superoxide dismutase; GSH-Px, glutathione peroxidase; CAT, catalase; MDA, malondialdehyde; T-AOC, total antioxidant capacity; ^a-c^Means within the same row with different superscripts are significantly different (*p* < 0.05).

### 3.5 Relative mRNA expression of Nrf2 and Keap1 in the liver

**Figure 1:**
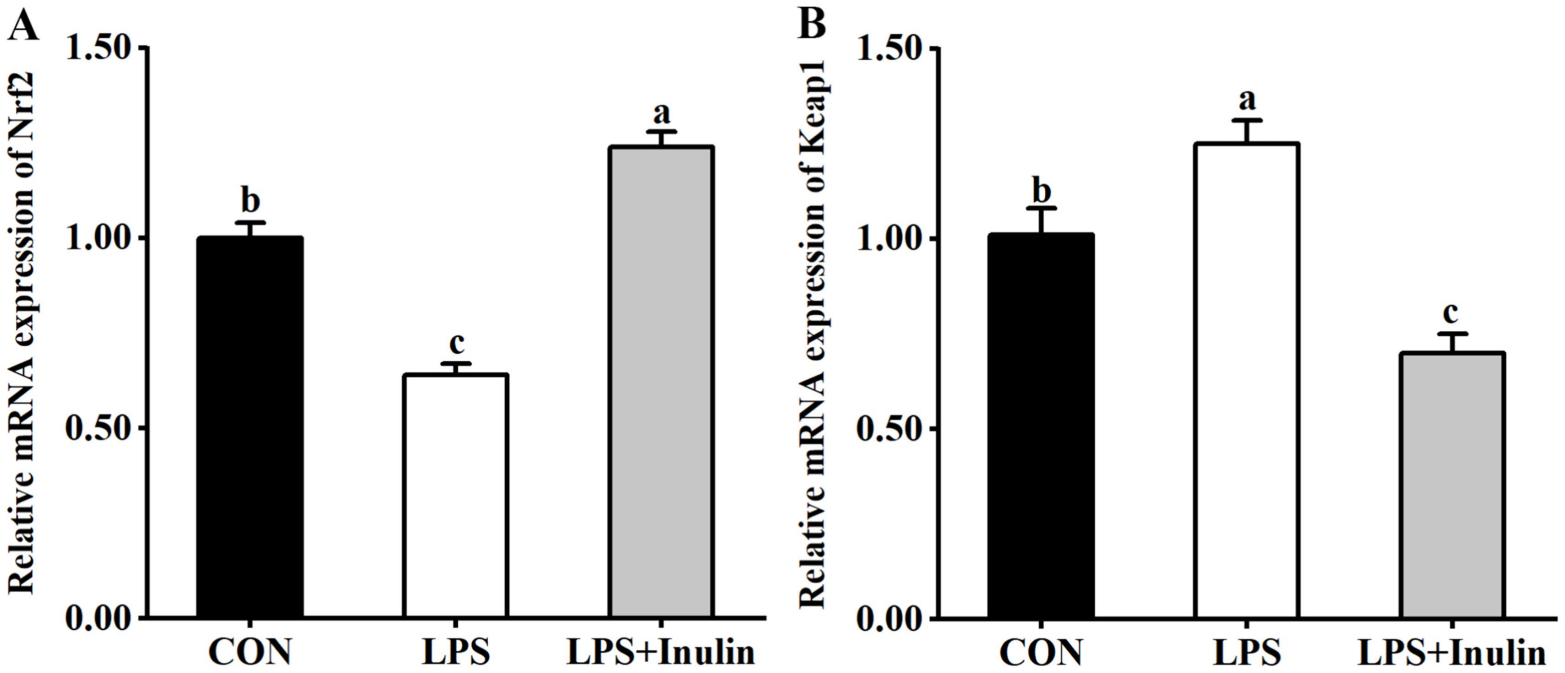
Effects of inulin on relative mRNA expression of Nrf2 **(A)** and HO-1 **(B)** in the liver of broilers challenged with LPS. CON, non-challenged broilers fed the basal diet; LPS, lipopolysaccharide-challenged broilers fed the basal diet; LPS + Inulin, lipopolysaccharide-challenged broilers fed the basal diet supplemented with 15 g/kg Inulin. Nrf2, nuclear factor erythroid-derived 2-related factor 2; Keap1, Kelch-like ECH-associated protein 1; Values are presented as mean ± standard error (*n* = 6). ^a-c^Means within the same row with different superscripts are significantly different (*p* < 0.05).

The effects of inulin on relative mRNA expression of Nrf2 and Keap1 in the liver of LPS-challenged broilers are presented in Figure 1. Significant down-regulation was found (*p* < 0.05) in hepatic Nrf2 mRNA expression after LPS injection compared to the CON group. Conversely, LPS administration significantly increased (*p* < 0.05) Keap1 mRNA expression relative to the CON group. Dietary supplementation of inulin significantly up-regulated (*p* < 0.05) hepatic Nrf2 mRNA expression and down-regulated (*p* < 0.05) hepatic Keap1 mRNA expression compared to the LPS or CON group.

## 4 Discussion

Oxidative stress is defined as an imbalance between the production of free radicals and the antioxidant capacity, which leads to cellular damage and tissue destruction (18). It has shown to play a pivotal role in the cascade of events underlying LPS-induced damage (16). The antioxidant properties of inulin have been documented *in vitro* and *in vivo* (12, 19). However, a critical knowledge gap persists regarding its capacity to counteract LPS-induced oxidative stress in broilers. Enzymatic antioxidant system, including SOD, GSH-Px and CAT, plays a crucial role in protecting various components of the body against oxidative stress (20). The T-AOC refers to the overall ability of the body to neutralize free radicals and combat oxidative stress. The MDA, as one of the terminal products of polyunsaturated fatty acid peroxidation, is widely recognized as a highly sensitive biomarker for evaluating lipid oxidative damage (21). As a result, this study systematically evaluated the effects of inulin on these antioxidant indicators across multiple tissues in LPS-challenged broilers.

It is widely acknowledged that serum biochemical indicators serve as critical markers of physiological and pathological states, which are essential for evaluating overall health (22). Consequently, serum antioxidant indicators were firstly determined. Previous studies have demonstrated that LPS stimulation could trigger systemic oxidative stress in broilers by reducing activities of antioxidant enzymes (e.g., SOD, GSH-Px and CAT), while simultaneously increasing MDA production (23, 24). Consistent with the previous results, the present study also found that LPS administration significantly decreased serum SOD, GSH-Px and CAT activities, and increased serum MDA production when compared to the CON group. The reduced activities of antioxidant enzymes and the elevation of peroxidation products further contributed to the significant decrease in T-AOC observed in the LPS group. Taken together, these results confirmed that LPS challenge compromised antioxidant capacity and induced systemic oxidative stress in broilers.

To date, few studies have focused on the effects of inulin on the antioxidant capacity of chickens under normal physiological conditions, and these studies indicate that inulin supplementation can enhance the chickens’ serum antioxidant capacity (12, 25). Similarly, our results also revealed that dietary inulin supplementation effectively prevented the LPS-induced reduction in serum SOD activity and the concomitant increase in serum MDA level. Although not significant, a marginal improvement was noted in serum GSH-Px and CAT activities by inulin supplementation. These ameliorations ultimately led to a significant increase in T-AOC in the LPS + Inulin group, though it remained lower than that of the CON group. These findings collectively suggest that inulin exerted protective effects against LPS-induced systemic oxidative stress in broilers.

Since serum indicators reflect the body’s oxidative stress status, the results of serum antioxidant parameters suggest that oxidative stress has occurred in certain tissues. The intestine is not only responsible for nutrient digestion and absorption, but also acts as the first line of defense against external pathogen invasion (26). However, due to its location at the interface between internal and external environments, the intestine is chronically exposed to Gram-negative pathogenic bacteria, making it more susceptible to LPS attack (7). Accumulating evidence has demonstrated that intraperitoneal LPS injection could induce intestinal oxidative stress in broiler chickens (8, 27). Consistently, in the current study, LPS administration also impaired jejunal antioxidant capacity and resulted in the development of oxidative stress as evidenced by reduced activities of SOD, GSH-Px, CAT and T-AOC and increased MDA level. Inulin has been reported to protect intestinal mucosa against oxidative stress induced by LPS in various species (28, 29). In the present study, supplementation of inulin partially eliminated the negative effects of LPS on SOD, GSH-Px and CAT activities, rendering them not significantly different from those in the CON group. In addition, dietary inulin supplementation significantly inhibited LPS-induced increases in MDA levels; however, MDA levels in the inulin-supplemented group remained higher than those in the CON group. Due to these above-mentioned improvements, inulin effectively enhanced jejunal T-AOC activity compared to the LPS group, restoring it to the level of the CON group. Overall, our findings indicated that inulin supplementation could alleviate LPS-induced oxidative stress in the jejunum.

Lipid oxidation constitutes a primary determinant of quality deterioration in chicken meat, which negatively influences its functional, sensory and nutritive values (30). Consequently, the antioxidant capacity of meat plays a crucial role in mitigating lipid oxidation, thereby maintaining meat quality and extending shelf life. It has been well documented that the antioxidant capacity of meat is susceptible to impairment under various stress conditions (31, 32). Our results also showed that LPS challenge weakened meat antioxidant capacity, as evidenced by the reduced SOD activity in both breast and thigh meat, and the elevated MDA level in thigh meat. The ability of inulin to improve antioxidant capacity in chicken meat has been demonstrated in a previous study (33). Consistently, this study found that supplementation of inulin partially mitigated the detrimental effects of LPS on SOD activity and MDA level, restoring both parameters to the levels of the CON group. These findings once again validate the role of inulin in enhancing muscle antioxidant capacity. The enhanced muscle antioxidant capacity by inulin supplementation is beneficial for improving meat storage stability and prolong its shelf life.

The liver, as a major site for reactive oxygen species (ROS) production due to its metabolic and detoxification activities, is continuously exposed to diverse toxic and reactive metabolites, rendering it highly susceptible to oxidative stress (34). Oxidative stress has been established as a common pathological mechanism involved in LPS-induced liver injury (15). Similarly, it has been reported that antioxidant enzyme activities, including SOD and CAT, were significantly decreased in the livers of broilers in response to LPS challenge (17). As expected, the current study revealed that LPS treatment hindered hepatic antioxidant capacity, as reflected by the decreased activities of SOD, CAT and T-AOC, alongside enhanced MDA production. It has been demonstrated that inulin exhibits antioxidant activity and hepatoprotective effects against liver injury, with its protective effects potentially attributed to the modulation of oxidative stress (35, 36). In the present study, inulin significantly prevented LPS-induced reduction in hepatic SOD and T-AOC activities, and slightly mitigated LPS-induced alterations in CAT activity and MDA level, suggesting the potential of inulin in alleviating LPS-induced liver injury.

To further explore the underlying molecular mechanisms by which inulin ameliorates LPS-induced hepatic oxidative stress, the relative mRNA expression of Nrf2 and Keap1 were evaluated. The Nrf2 pathway is a crucial redox-responsive antioxidant defense mechanism, regulating the expression of cytoprotective genes to combat oxidative stress and maintain cellular homeostasis (37). Under normal conditions, Nrf2 is inactive in the cytoplasm owing to its binding to Keap1. Upon oxidative stress, Nrf2 dissociates from Keap1, translocates to the nucleus, and regulates the transcription of antioxidant enzymes involved in the scavenging of ROS and other oxidants (38). It has been reported that LPS injection down-regulated the mRNA expression of Nrf2, and up-regulated that of Keap1 in the liver of broilers (15, 23). In agreement with the above, the present study also found that LPS inhibited the Nrf2 signaling pathway by suppressing Nrf2 expression and enhancing Keap1 expression in the liver. Previous research has proved the ability of inulin in preventing high-fat-induced decrease in the hepatic Nrf2 expression of mice (39). The current study revealed that dietary inulin supplementation elicits a coordinated regulation of the hepatic Nrf2/Keap1 axis, as evidenced by the significant upregulation of Nrf2 mRNA expression and concomitant downregulation of Keap1 mRNA expression. These results suggest a mechanistic role for inulin in mitigating LPS-induced oxidative stress, potentially by activating the Nrf2-mediated antioxidant response pathway.

## 5 Conclusion

In conclusion, the present study revealed that LPS challenge compromised antioxidant capacity in multiple tissues including liver, intestine, and muscle, thereby resulting in systemic oxidative stress. Dietary inulin supplementation could effectively alleviate LPS-induced oxidative stress putatively through regulation of the Nrf2 signaling pathway.

## Data Availability

The original contributions presented in the study are included in the article/supplementary material, further inquiries can be directed to the corresponding author.
